# Observations on Syncope Angens

**Published:** 1817-03

**Authors:** J. Johnson

**Affiliations:** St. George's Square, Portsea


### *OBSERVATIONS on SYNCOPE ANGENS.*

By Dr. Johnson.

( Continued from page 108. )

IN the foregoing Case there is one remarkable peculiarity, which, I believe, has not been observed in any other instance of angina pectoris, namely, the circumstance of one side of the heart being nourished with blood, while the other might be compared to Tantalus dying with thirst, yet surrounded with drink! The left side of the heart was continually called upon to supply the whole arterial system with blood, yet had its own proper channel of supply cut off. In what way then was it nourished? Only by the scanty regurgitation, along its coronary artery, of blood from inosculations with its fellow of the right side. This accounts for the trunk and branches of the left coronary keeping open, though diminished in calibre, after the *origin* of the vessel was obliterated.

The left side of the heart was not only deprived of its nutrient blood, and thereby weakened; but its function was impeded by the dilatation and rigidity of the aorta, in consequence of which, the semilunar valves were incapable of preventing a portion of blood from rushing back into the ventricle during each diastole of the heart. The effect of both these causes operating in a similar manner, must have been, that the left side of the heart was unable to propel the blood into the general system as fast as it returned from the pulmonary; hence a congestion of blood in the lungs and about the left auricle at all times; (the habitual dyspnœa); and when, by any of the exciting causes, the paroxysm was induced, that dreadful sense of suffocation, pain, and stricture about the region of the heart. These causes continuing and increasing, the passive dilatation of all the cardiac cavities was a matter of course, and cannot excite surprise. How far the extensive, and nearly inseparable adhesions of the lungs to the parietes of the thorax, especially on the right side, were caused by the obstructed circulation in the pulmonary system, may be a matter of doubt; as he declared that, till the present complaint occurred, he never had an hour's sickness in his life, nor ever, as far back as he could remember, took a particle of medicine. The adhesions alluded to, however, might contribute to that habitual dyspnœa and slight wheezing which he experienced between the paroxysms.

The pain and sense of numbness, felt pretty generally in the left arm in angina pectoris, are perfectly inexplicable; as are those various other pains and morbid sensations complained of in different and frequently remote parts of the body, when the central organ of circulation is diseased. In this man the kidnies were often, but the left arm always, the seat of pain; and it is not a little remarkable, that the first attack of the complaint commenced with pain about the insertion of the deltoid muscle, which immediately darted into the left breast. Why this pain and cramp should creep down along the ring and little finger, never along the others, is also a puzzling question. There can be little doubt but the anomalous and wandering pains, felt in remote parts of the human frame during the advance of cardiac diseases, have often drawn off the attention both of the patient and practitioner from the original malady till it was too late, or led to remedies sometimes prejudicial to the real disorder. Now, on the other hand, they serve as guides, or Ariadne's clue, in the investigation of diseases of this class.

The relief experienced from the erect posture, and from raising the arms above the head, clinging to a post or door, &c. may be satisfactorily accounted for by their effects in dilating the chest, elevating the ribs, and probably by bringing the arch of the aorta into a straight line. The circumstance of the patient's always endeavouring to make a deep inspiration, and retain the air in the lungs as long as possible, admits of a similar explanation.

In what way the flatulence of the stomach is connected with cardiac derangement, would be difficult to conjecture; but the connexion is most certain, and the relief obtained from expelling flatus is very evident. This last is easily enough accounted for, by the mechanical pressure which an inflated stomach must make on the sphere of the heart's action. The remarkable distention of the stomach and intestines by air, in Myrtle's case, is not singular, as the circumstance has been often remarked before in others.

That the hæmorrhoidal discharge should be attended with mitigation of the paroxysms may not surprise us; but that a constipated state of the bowels should be found more favourable to the complaint (as the patient assured me was the case), is unaccountable on any other supposition than that *straining* at stool, during intestinal relaxation, should increase the disposition to attack, as has been remarked by many writers on the disease in question. In this man's case, a griping stool almost always brought on more or less pain in the region of the heart.

The inequality of the pulse in the two arms, and the want of synchronous action between the heart and the radials, were ascertained by Dr. Denmark and myself during a paroxysm of the disease, in which we one day happened to find him. It was also confirmed by his own testimony, and that of another person who used to feel his pulse at those times. I may remark, however, that he never, till the hour of his death, experienced what may be strictly termed *syncope*, and that in the worst paroxysm in which I saw him, and finally in the paroxysm which terminated his existence, the pulse never ceased, nor even became notably weak, till it ceased for ever. In the last paroxysm, my assistant particularly examined the pulse, as I had made him acquainted before with the nature of the disease; and on returning to me, an hour before the patient's death, he reported, that the pulse was neither intermittent nor particularly weak. Whether the singularity attending this case of angina pectoris, namely, the obliteration of only *one* of the coronary arteries, may have produced a deviation from the more general train of phænomena in this point, I do not pretend to determine. He stood up during the last paroxysm, leaning on a door, till within half a minute of his death; in short, he sat down on the bed-side and instantly expired.

Perhaps it may not be unprofitable to offer a conjecture on the probable relative progress and comparative priority of the morbid phænomena.

Whether the fall and contusion on the region of the heart, sustained some years previously to the development of angina pectoris, may have laid the foundation for the succeeding train of fatal disorganization, is a matter of the greatest uncertainty; yet I am inclined to think they might have had some share in giving origin to what I conceive to be the first structural derangements which took place—the enlargement and induration of the aorta. The dilatation in the calibre, and the cartilaginous and osseous depositions in the coats of the artery, clearly prove that these derangements must have been going on long before the symptoms of angina pectoris appeared. Synchronous, and probably *pari passu* with induration and dilatation of the aorta, was the march of passive enlargement of the cardiac cavities. The reflux of blood from the inelastic aorta, would throw a disproportionate duty on the left; the remora of blood in the lungs, on the right side of the circulating organ; both tending to produce, in a weak heart, passive dilatation. But the aortic induration, while it caused the above-mentioned derangements of structure and function in the heart, was silently laying the foundation for a still more terrible lesion. It was gradually closing up the origin of that channel which conveyed nutrition to one half of an organ already embarrassed and overstrained. I should conceive it probable, that the progressive exasperation of the angina pectoris was commensurate with, and dependant on, the advancing obliteration of the origin of the coronary artery; and that shortly after the total obliteration, the left side of the heart was incapable of performing its duty. What was now the consequence? The right side of the heart, though thin and dilated, was still nourished by its proper artery, and capable of driving the blood into the lungs; while the left side, embarrassed with a dilated aorta, attenuated in its muscular parietes, dilated in its cavities, and unnourished by its nutrient vessel, could no longer transmit the blood from the lungs to the body, but died in the unavailing effort of disgorging the load with which it was oppressed!

*Prevention, Relief, Cure, &c.*

It is almost needless to speak of the prevention of syncope angens, since we are ignorant of the means which can retard ossification in the sanguiferous system. Dr. Parry, indeed, hazards an opinion that these ossific depositions depend on "an increased impetus of the blood, more especially when amounting to inflammation;" and with this view, recommends temperance in eating and drinking; abstinence from violent bodily exertion; and, in short, an attention to all the antiphlogistic measures which experience has pointed out as efficacious in arresting the progress of inflammation in general. It is well known, however, that this rule comprehends within it the plan of avoiding all those exciting causes by which the paroxysms of syncope angens are brought on, and therefore we may think that we are checking the organic derangement, when we are only warding off those circumstances that make its morbid influence more apparent. Still we are, in so doing, acting on the safe side.

With respect to a *cure*, although some instances are on record, we have reason to be very sceptical on this head. Dr. Parry says, that one of his patients, who had laboured under angina pectoris, "though considerably advanced in years, is now (by the Bath waters) wholly, or nearly free from the disease." 159. Does Dr. Parry think the disease, in this case, was ossification of the coronary arteries? Is it probable, or even possible, that any human means could have opened again, in my patient, the origin of the left coronary artery, after it was sealed over by the cartilaginous and ossific deposition on or between the coats of the aorta?

The most circumstantially related cure of angina pectoris by the nitrate of silver, is in the case of John Maynard, by Dr. Robert Cappe, in the fourth vol. of the Med. and Phys. Journal. But there are many circumstances in this case which invalidate the probability of its being genuine angina pectoris, and particularly ossification of the coronary arteries. The "cough, spitting of blood, and pain in his *arms*," near the insertion of the deltoid muscles, are not essential to angina pectoris though they may indicate *some other* thoracic derangement. Neither is the "inexpressible sensation of severe pinching at the pit of his stomach" at all a symptom of genuine angina pectoris. Moreover, "on pressing on the *right side* below the ribs, near the scrobiculus cordis, he feels pain. This is the seat of his peculiar sensation." Again, "he has a constant cough with very little expectoration," and "he has never had the sensation of his heart ceasing to beat—it cannot be felt in any part of the thorax." These extracts evidently prove that the complaint of Maynard was any thing but angina pectoris, and may be classed under the Italian Professor's head of "*Steno-cardia*," as there is strong reason to believe that the liver was affected; and from the *intermitting* pulse, there was probably effusion in some of the thoracic cavities.

An interesting case of angina pectoris is related by my friend, Dr. Palmer, in the eighth vol. of the *New Med. and Phys Journal;* wherein "great relief" was obtained by instituting a permanent drain from the region of the heart, abstinence, quietude of mind and body, moderate exercise in the open air, antiacids, bitters, aromatics, laxatives, &c. Yet, here there is no proof of any thing like a cure of the disease. The paroxysms only were warded off. "He can never deviate," says Dr. Palmer, "from it (the plan detailed) in any considerable degree, with impunity." P. 449.

Where the coronary arteries are ossified or cartilaginous, but still pervious, however small their remaining calibre, some hope may be entertained, by extreme abstinence and quietude, of procrastinating death and mitigating the attacks; but, where the source of nutrition is once closed up, as in the case of my patient, no human power can prevent the fatal catastrophe.

In respect to the treatment during the paroxysm itself, I think the case in point offers an instructive lesson. One of the coronary arteries obliterated; the cavities of the heart dilated, and the parietes attenuated, pale, and flabby, offer so *apparently* insuperable objections to blood-letting, that a decisive proof of its utility in *such* a case may be ample authority for carrying it to a farther extent on similar occasions in future, than is recommended by the best writers on the subject. From Dr. Parry's patient, Mr. S. nine ounces of blood were abstracted in the paroxysm, during which "*the pulse became fuller and stronger;* he could not, however, perceive that his pain was, in any degree, alleviated." P. 20. Now, at the time that I was bleeding my patient, in the first, and by far the most violent paroxysm in which I saw him, I did not know that the disease was angina pectoris, and as the relief of pain was my object, I went on with a fortunate temerity till sixteen—twenty—twenty-four ounces were abstracted, by which time almost every symptom of the paroxysm disappeared, the *pain* being the last to vanish. Had I been aware of the real nature of the disease, I should have stopped short, with a partial relief of the symptoms, or rather, I am disposed to think, death would have closed the scene, for it is impossible he could have lived many minutes more, had not effectual relief been procured.

Every circumstance in the pathology of angina pectoris evinces that the paroxysm is brought on rather by an unusual accumulation of blood about the heart, than by any *unusual* weakness of the organ at the moment. Thus the exertion of walking up stairs can produce no immediate debility of the heart; but it can produce an *inordinate* afflux of blood to the organ, and thereby overpower it. What then is to be done? Is the phantom *debility*, or is the fear of *weakening* the heart itself, to prevent us from drawing off the load under which it lies suffocated? Dr. Percival informs us, that "nothing afforded such instantaneous ease during the paroxysms of this disorder as venæsection and vomiting." We know that vomiting most powerfully diffuses the blood from the centre to the surface of the system; but before the action of emetics can be brought on, death may take place. Venæsection itself very frequently produces nausea and vomiting, and appears to be the first remedy that should be tried, though it does not preclude others. Dr. Parry justly remarks, that "the extreme weakness of the pulse, and coldness of the skin, certainly do not contra-indicate bleeding." P. 162. He illustrates this by the case of Sir E. W. who had, for many years, suffered severely from palpitation of the heart, attended with difficulty of breathing and a sense of immediate suffocation; during which, his pulse was extremely weak, his face and extremities livid, and bathed in a cold sweat. In two such attacks, he was relieved by bleeding. "In a third paroxysm of the same kind," says Dr. P. "a happy opportunity occurred of comparing the effect of this remedy with that of a *stimulant* taken into the stomach. The apothecary, who had reached the patient before me, while I was occupied in feeling the pulse, took up a cordial mixture which stood on the table, and gave some spoonfuls of it to the patient. In a few seconds, the pulse rapidly sunk, so as scarcely to be felt; and the coldness and dyspnœa increased in so remarkable a degree, that we could scarcely flatter ourselves that the patient would live many minutes. A vein was now opened, and, as on former occasions, before three ounces of blood had flowed, the just fullness of pulse returned, and the breathing became perfectly natural." P. 164.

Dr. Parry thinks that in the paroxysms of angina pectoris blood should be taken from a small orifice, the patient being placed in the horizontal position, while the physician is to keep his finger on the pulse, to decide on the limits to which venæsection is carried. I think the foregoing case will shew that little danger is to be apprehended from a free evacuation of blood upon these occasions.

There is hardly any other remedy which, during the paroxysm itself, can be relied on as offering any effectual relief. In such cases, where death-like coldness and debility characterise the morbid state, and pressing danger, it is natural enough to fly to cordials; but their mischief has already been pointed out, and therefore they should be used with great caution, and only to remove flatulency from the stomach. Dr. Parry suggests rubefacients, frictions, and external heat to the lower extremities as preferable to stimulants. Would not the tepid bath, by eliciting the blood to the surface, prove useful in relieving the central organ of circulation from the load with which it is oppressed?

J. JOHNSON, M. D.

St. George's Square, Portsea,
September, 1816.

PART

